# Anti-biofilm activity of marine algae-derived bioactive compounds

**DOI:** 10.3389/fmicb.2024.1270174

**Published:** 2024-04-12

**Authors:** Asma Behzadnia, Marzieh Moosavi-Nasab, Najmeh Oliyaei

**Affiliations:** ^1^Seafood Processing Research Center, School of Agriculture, Shiraz University, Shiraz, Iran; ^2^Department of Food Science and Technology, School of Agriculture, Shiraz University, Shiraz, Iran

**Keywords:** anti-biofilm activity, algae extracts, biofilm, marine natural products, quorum sensing, quorum quenching, quorum sensing inhibition

## Abstract

A large number of microbial species tend to communicate and produce biofilm which causes numerous microbial infections, antibiotic resistance, and economic problems across different industries. Therefore, advanced anti-biofilms are required with novel attributes and targets, such as quorum sensing communication system. Meanwhile, quorum sensing inhibitors as promising anti-biofilm molecules result in the inhibition of particular phenotype expression blocking of cell-to-cell communication, which would be more acceptable than conventional strategies. Many natural products are identified as anti-biofilm agents from different plants, microorganisms, and marine extracts. Marine algae are promising sources of broadly novel compounds with anti-biofilm activity. Algae extracts and their metabolites such as sulfated polysaccharides (fucoidan), carotenoids (zeaxanthin and lutein), lipid and fatty acids (γ-linolenic acid and linoleic acid), and phlorotannins can inhibit the cell attachment, reduce the cell growth, interfere in quorum sensing pathway by blocking related enzymes, and disrupt extracellular polymeric substances. In this review, the mechanisms of biofilm formation, quorum sensing pathway, and recently identified marine algae natural products as anti-biofilm agents will be discussed.

## 1 Introduction

Microbial sessile communities attached to a substratum or interphases or to each other, known as biofilms embedded in self-secreted extracellular matrices containing protein, polysaccharide, nucleic acid, and lipid substances, display an altered phenotype in comparison with planktonic microbial cells (Muras et al., 2021; Wang et al., 2022). Extracellular polymeric substances (EPS) is greatly involved in the stability of the structure and the function of biofilm (Ashrafudoulla et al., 2020). Over 80% of microbial infections are associated with biofilm formation, while it has been characterized as one of the crucial medical hurdles over the century (Brackman and Coenye, 2015; Wang et al., 2022). Moreover, bacteria cells within biofilms have shown 1,000 times more resistance to various stresses rather than the planktonic form (Ashrafudoulla et al., 2019). In addition to their impact on human health, biocorrosion and biofouling based on biofilm formation of microorganisms cause enormous financial problems in the shipping and medical industries (Muras et al., 2021; Wang et al., 2022). Therefore, due to the importance of this issue, some researchers present multiple challenges of biofilm formation and their impact on food safety, particularly poultry and seafood products (Ashrafudoulla et al., 2023; Chowdhury et al., 2023), and fully discuss the new insights to combat biofilms in the food industry (Mevo et al., 2021; Rahman et al., 2023; Uddin Mahamud et al., 2024). In recent years, numerous natural products have been identified as antibacterial and anti-biofilm agents (Song et al., 2018; Lu et al., 2019; Mulat et al., 2022) which can inhibit cellular adhesion, abolish the quorum sensing signaling, prevent biofilm formation, disrupt extracellular matrix (ECM) structure, and decrease the production of quorum sensing-regulated virulence factors in pathogenic bacteria (Lu et al., 2019). Therefore, several research studies have been focused on natural anti-biofilm agents obtained from plants, microorganisms, and marine extracts or metabolites to control human infections (Mishra et al., 2020; Danquah et al., 2022; Ashrafudoulla et al., 2023). Among various natural products, it is well known that marine and algal bioactive compounds are utilized for prevention and treatment of bacterial biofilm (Melander et al., 2020).

Algae are known as renewable sources of bioactive compounds, in particular, carotenoids and polysaccharides, with a wide range of biological activities, such as antioxidant, antimicrobial, anti-inflammatory, anti-obesity, antidiabetic, anticancer, and anti-Alzheimer activities (Oliyaei et al., 2023). Macroalgae or seaweeds are classified into Chlorophyceae (green algae), Phaeophyceae (brown algae), and Rhodophyceae (red algae) (Oliyaei et al., 2022). Some research studies revealed that their extracts or metabolites have the potential to inhibit biofilm formation (Zammuto et al., 2022). Similarly, microalgae, microscopic and photosynthetic organisms, are rich in various metabolites (polyphenols, lipids, and carotenoids) with various bioactivities. Microalgal derivatives are promising candidates in novel, biocontrol agents against pathogens (Sampathkumar et al., 2019).

Based on the importance of biofilm, and concern about its serious problems, natural substances became attractive. Moreover, considering the potential of the use of algae as a novel source of biological agents, determining the antibiofilm activities of their derivatives is crucial. To the best of our knowledge, there is no review article about the anti-biofilm inhibitory potential of different algal-based bioactive compounds. Therefore, this review summarizes an overview of biofilm formation, quorum sensing pathway, and new identified marine algae natural products, as anti-biofilm agents will be discussed. These natural substances are interesting candidates that suggest novel strategies for controlling biofilm-related infections.

## 2 Biofilm formation and structural and physiological attributes

**Figure 1:**
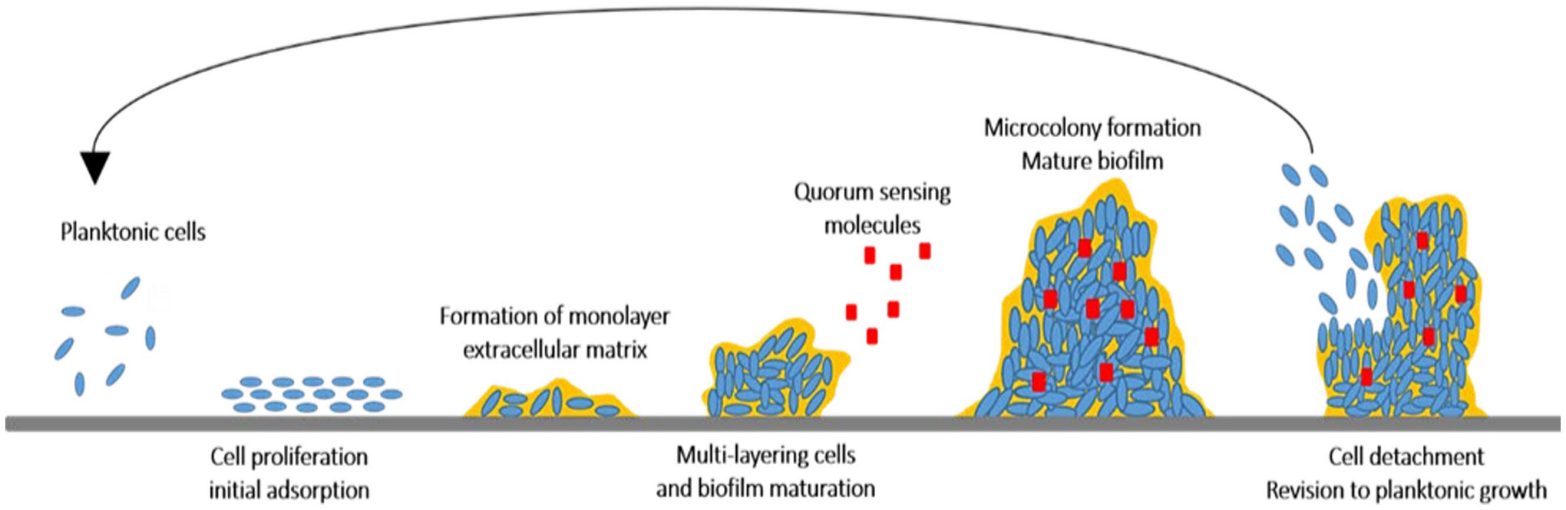
The cycle of biofilm evolution from planktonic cells to detachment of planktonic cells.

Biofilm is a sessile complex of microorganisms produced on food surfaces. Biofilms create serious concerns in the healthcare and food industries. The structure complexity of biofilms causes intrinsic resistance against antibiotics and also promotes the colonization of microorganisms by providing nutrients which protect microbial cells from antimicrobial agents (Ashrafudoulla et al., 2021). Biofilm occurrence forms in multiple steps as shown in Figure 1: (i) conditioning the surfaces with organic and inorganic molecules influencing initial adherence of the microbial cells and (ii) producing extracellular matrices causing attachment of cells to the substratum more firmly. This allows the cells to grow and mature from microcolonies to three-dimension communities. The physiological responses of the microbial cells in the biofilm community will change based on the existing conditions in the biofilm, (iii) detaching the cells from the biofilm, disperse and form a biofilm on other surfaces or release as a planktonic state (Brackman and Coenye, 2015; López and Soto, 2019).

A biofilm association is evaluated as one of the most impressive sites of genome expression of bacterial cells, in which the microbial cells are well preserved and metabolically act more efficiently. However, a biofilm arrangement with attributes, such as survival, adaptive, and protective values, behaves differently from the planktonic cells that are not very efficient and are only used for propagating and colonizing new surfaces. The population of microbial cells embedded in the biofilm matrix is between 10^4^ and 10^8^ cells/cm^2^, by which the differences between invisibility and visibility of biofilms are being considered (Costerton et al., 1995; Lazar, 2011; Lamin et al., 2022).

Moreover, a biofilm community resists microbial cells inside the matrices to antibiotics by numerous mechanisms. The small dissemination of antibiotics across the extracellular matrices of biofilm communities, altered physiological traits of microbial cells by the low growth rate in response to restrict oxygen and nutrient quantity or environmental stress, changed phenotypic properties of cells, quorum sensing. Then, it results in exchanging the resistance of genes between microbial cells inside the biofilm and resisting the cells exposed to antimicrobial agents due to the remaining cells and cell debris. In addition, the structure of the biofilm would be effective in its mechanism of antimicrobial resistance which avoids the immune system function and phagocytosis (López and Soto, 2019). A large number of microbial species tend to communicate with other cells, make an interaction with their surrounding environment, and display multicellularity, resulting in biofilm formation (Kalia et al., 2015; Muras et al., 2021; Majdura et al., 2023). It is mostly related to the ability of microbial cells to communicate with themselves at high and uncommonly low cell densities through the signaling phenomenon called quorum sensing. Quorum sensing system was utilized to synchronize gene expression to biofilm formation, indicating cell density and facilitating collective behaviors. Cell-to-cell communication known as quorum sensing has been found to be crucial in biofilm formation of both gram-negative and gram-positive with the surrounding extracellular matrix. By detecting and quantifying the accumulation of specific self-produced signal molecules released by the community, quorum sensing allows bacteria to determine the population density. The quorum sensing system mediates changes in the production of biofilm matrix components, such as exopolysaccharides, lipids, nucleic acids, and proteins. In addition, this system is also coordinated with other environmental factors such as pH, salinity, temperature, oxidative stress, and availability of nutrients progressing the cellular adaptation to the environment and enhancing their survival likelihood. However, interfering with the quorum sensing system is known as a promising approach to avoid bacterial biofilm proliferation (Machado et al., 2020; Muras et al., 2021).

## 3 Quorum sensing signaling network

As mentioned above, there is a cell-to-cell communication among the microbial cells involved in the biofilm matrix. Quorum sensing is mediated by the production and secretion of low molecular weight chemicals, also known as auto-inducers (AIs) (Menaa et al., 2021), using bacterial cells depending on their cell density (Lazar, 2011; Machado et al., 2020). These diffusible signaling molecules are responsible to regulate gene expression depending on cell density and to adjust some prokaryotic phenotypes such as bioluminescence, production of secondary metabolites (toxins and antibiotics), secretion of intercellular enzymes, production of virulence factor, and biofilm formation (Borges and Simões, 2019; Lamin et al., 2022). Over the last years, the function of the quorum sensing system has been investigated in several important biological processes. It has been shown that quorum sensing mechanism regulates food spoilage. In addition to regulating gene expression, it has also an impressive effect on the survival and growth of food pathogens (Bai and Vittal, 2014; Machado et al., 2020). Studies have demonstrated the crucial role of the quorum sensing process in different stages of biofilm formation (Machado et al., 2020). The most identified microorganisms involved in biofilm formation mediated by quorum sensing signaling network include *Pseudomonas aeruginosa*, *P. fluorescens*, *Chromobacterium violaceum*, *Campylobacter jejuni*, *Aliivibro ficheri*, *Vibrio harveyi*, *V. parahaemolyticus, Escherichia coli*, *Acinetobacter* spp., *Salmonella enterica*, and *Salmonella* Thompson. They are known as human opportunistic pathogens causing infections such as pneumonia, chronic otitis, chronic wounds, and catheter infection (Ashrafudoulla et al., 2019, 2023; Ćirić et al., 2019).

**Figure 2:**
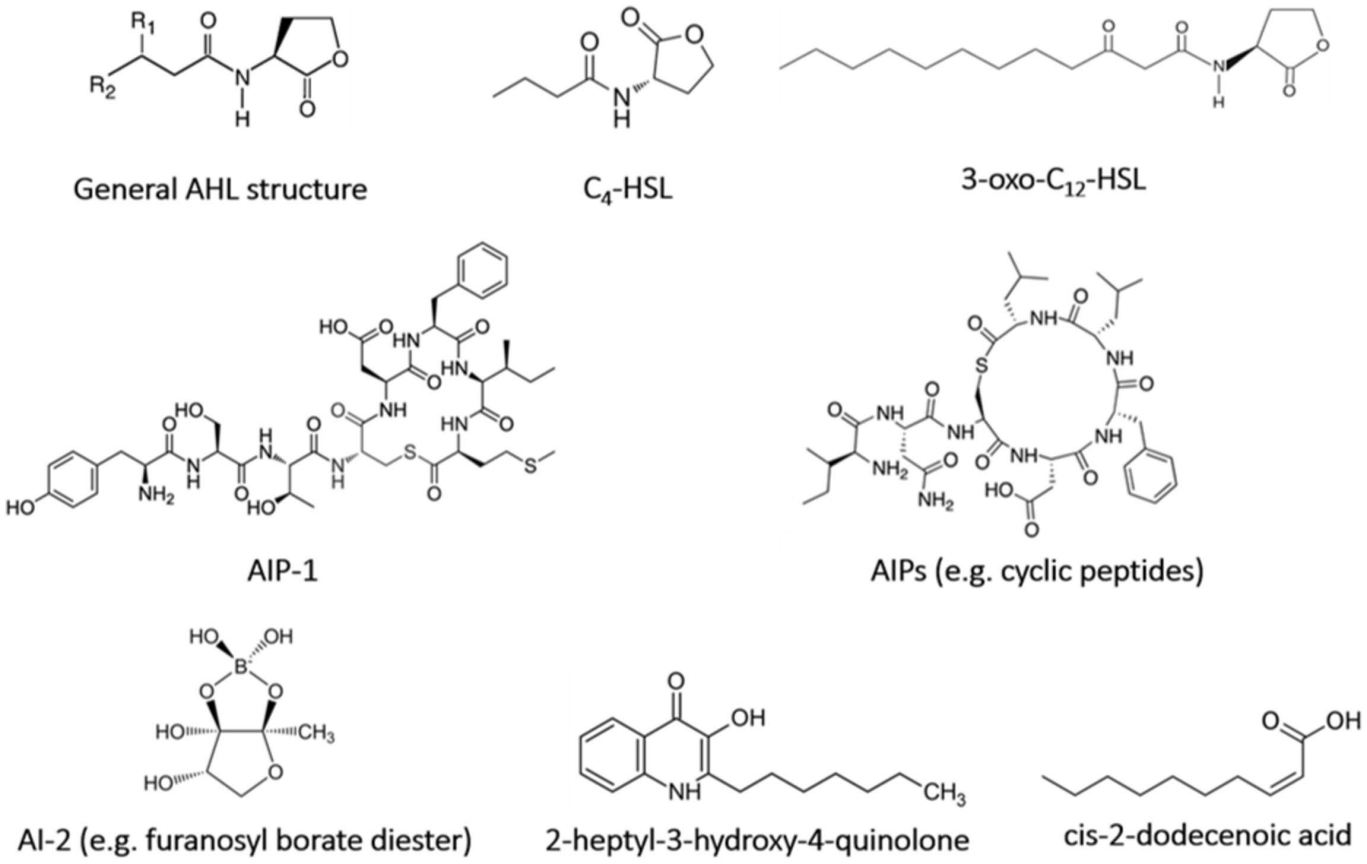
Chemical structure of quorum sensing signal molecules.

However, quorum sensing signaling systems can be used in expanding quorum sensing inhibitors to control the biofilm propagation, specifically unwanted bacterial embedding in the biofilm matrix (Machado et al., 2020). The auto-inducers (AIs) are synthesized steadily and diffused into the environment with the cell density increment gaining a threshold value called quorum level of signaling molecules (Borges and Simões, 2019; Machado et al., 2020). Different types of quorum sensing signaling systems (Figure 2) distinguished between gram-negative bacteria cytoplasm and gram-positive bacteria cell membrane, exploiting the gene regulation-mediated quorum sensing (Borges and Simões, 2019). Hormone-like AIs diffusible from gram-negative bacteria, such as acyl-homoserine-lactones (AHL), non-diffusible autoinducing peptides (AIP) at gram-positive bacteria, and autoinducer-2 (AI-2) in both gram-positive and gram-negative bacteria are major quorum sensing systems which are recognized as precursor peptides releasing outside by protein transport machinery (Lazar, 2011; Brackman and Coenye, 2015; Borges and Simões, 2019). Moreover, the other typical signaling molecules such as *Pseudomonas* quinolone signal (2-heptyl-3-hydroxy-4-quinolone), fatty acids (cis-2-dodecenoic acid), and autoinducer-3 (from *E. coli* O157:H7) have been identified, while the LuxI/R-type system, the Agr system, and the LuxS/AI-2 system are known as the main quorum sensing systems (Bai and Vittal, 2014; Borges and Simões, 2019). The LuxI/R-type system consists of the proteins called *LuxI* (AHL synthase) and *LuxR* (AHL receptor), utilized by gram-negative bacteria. The regulation of the luminescence production in gram-negative marine bacterium *V. fischeri*, which is known as the cell density-dependent mechanism, is mediated by the AHL quorum sensing system (Papenfort and Bassler, 2016; Borges and Simões, 2019). The Agr system consists of RNA II and RNA III regulating signals. It is a signaling peptide presenting in gram-positive bacteria such as *Staphylococcus aureus* as activating/inhibiting peptides that contain a membrane-bound sensor and an intracellular response regulator (Brackman and Coenye, 2015; Vadakkan et al., 2018; Borges and Simões, 2019). The third signaling system is the LuxS/AI-2 system which is associated with the AI-2 production mediating the interaction between gram-negative and gram-positive bacteria (Brackman and Coenye, 2015). The LuxS protein is key a metallo-enzyme with two tetrahedral metal-binding sites being in some bacterial species, i.e., *Streptococcus* spp., *Lactococcus lactis*, *Clostridium perfringens*, *Neisseria meningitidis*, *E. coli*, and *Haemophilus influenza* (Brackman and Coenye, 2015; Wang et al., 2018).

However, it has been demonstrated that biofilm spoilage related to the quorum sensing network is highly responsible for contaminating various food products such as milk and dairy, meat, fisheries, and vegetables products (Machado et al., 2020; Lamin et al., 2022). For instance, *Salmonella* spp., *Campylobacter* spp., *Listeria monocytogenes*, *E. coli*, *S. aureus,* and *Bacillus cereus* as foodborne pathogens are capable to connect different surfaces in various industries causing biofilm distribution, which would be a severe health and hygiene problem and an economic problem (Giaouris et al., 2015). Therefore, since there are still more to learn about the key role of quorum sensing in biofilm formation, antagonist molecules, known as quorum sensing inhibitors/quorum quenching agents by binding to different sites on the receptors (LuxR-type receptor), block the signal activations. The blocking agents compete or interfere with AHL signal molecules, target production of cell signaling molecules, deteriorate signal systems, and inhibit the attachment of signals to the receptors or signal transportation to prevent the biofilm formation (Bai and Vittal, 2014; Brackman and Coenye, 2015). However, quorum sensing inhibition as an alternative strategy might result in the inhibition of particular phenotype expression blocking cell-to-cell communication that would be more acceptable rather than conventional sanitizers (Muras et al., 2021).

## 4 Quorum sensing inhibition

**Figure 3:**
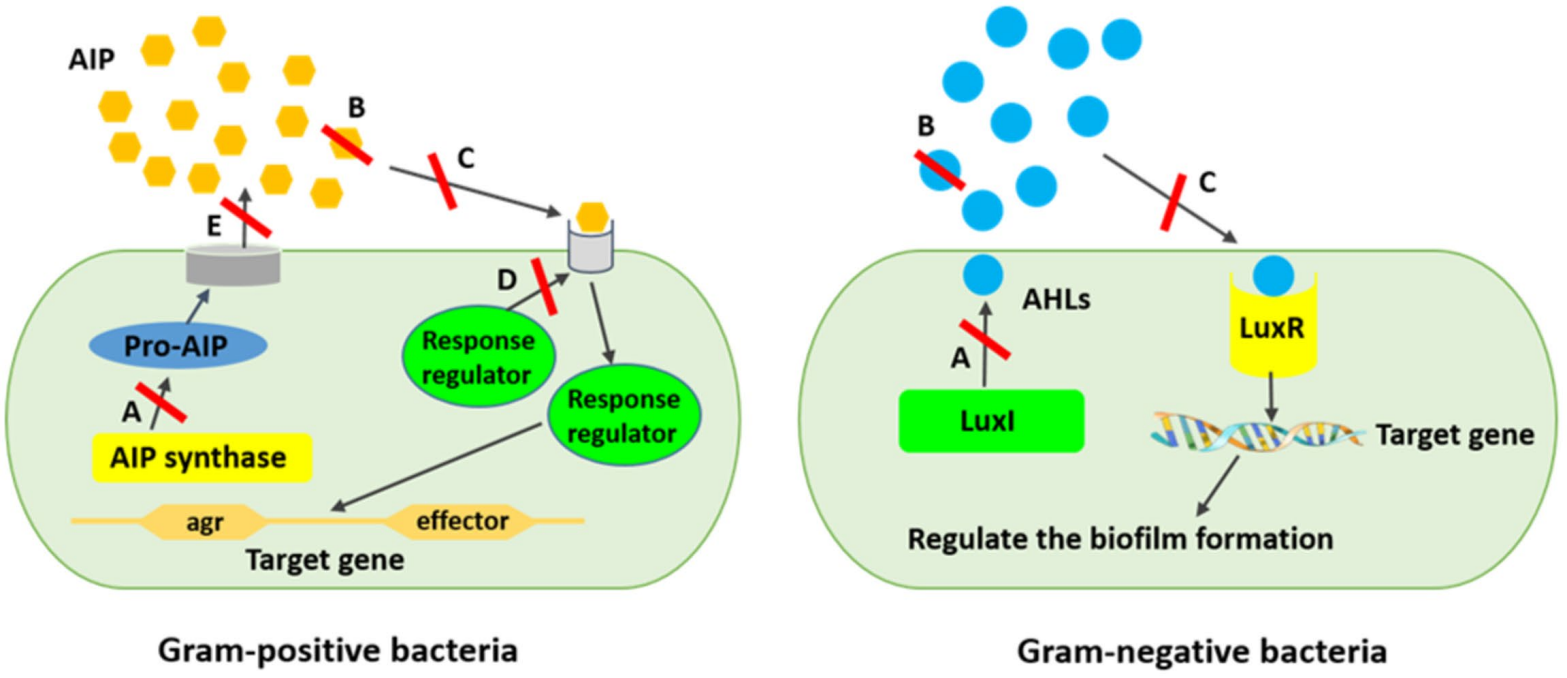
Mechanism of quorum sensing inhibition against biofilm formation. **(A)** AIs synthesis inhibition, **(B)** Degrading AIs by AHL-lactonases, **(C)** Interference of the signal receptors by AI antagonists, **(D)** Interference of the response regulators resulting in signaling cascade disturbing, **(E)** Reduction of the extracellular AIs resulting in inhibited cell-to-cell signaling. *AIPs are produced inside the bacterial cell as pro-AIP which will be processed and modified inside or outside the cell hinge upon the organism.

Several strategies have been expanded to eradicate quorum sensing network and biofilm-related infections. Applying liposomes, bacterial interference, bacteriophages, hydrogels, iontophoresis, and nanoparticles are some approaches combatting biofilm propagation, although new approaches are also required (López and Soto, 2019). Therefore, the inhibition of the quorum sensing network using new materials and strategies is a promising tool for interfering bacterial biofilms, while they resist to a variety of regular antimicrobial agents (Lazar, 2011; Kalia et al., 2015). The polymeric matrix on the surface of the substratum, which is mostly made up of exopolysaccharides, extracellular proteins, nucleic acids, and other components, facilitates the irreversible adhesion of bacteria. Cell adhesive properties also depend heavily on extracellular elements, such as surface-exposed proteins, extracellular glucan-binding proteins, and glycosyltransferases. Hence, the anti-biofilm function of natural compounds is related to the blockage of polymer matrix synthesis, elimination of cell-to-cell and cell-to-surface attachment, extracellular matrix disruption, lowering of the formation of virulence factors, and, subsequently, hampering quorum sensing system and biofilm formation (Lu et al., 2019). Quorum sensing inhibition or quorum quenching mechanism is referred to the enzymatical inactivation of quorum sensing signals. It describes the disruption of microbial cell communication by quorum sensing inhibitory chemicals using targeted to AI signaling molecules, receptors, and downstream signaling cascades (Figure 3) (Lazar, 2011; Kalia et al., 2015; Muras et al., 2021).

It has been reported that blocking the microbial quorum sensing signals as a prevalent strategy has already been applied by a variety of organisms to protect themselves from pathogenicity and virulence (Lazar, 2011; Sampathkumar et al., 2019). This strategy inhibits intercellular signaling by (i) hindering diffusion of homoserine-lactone (HSL) signal and lowering the concentration of extracellular HSL. Signal molecules are degraded by enzymes such as AHL-lactonases and AHL-acylases, which hydrolyze the lactone ring and release free HSL and fatty acid, respectively; (ii) inhibiting the signal receptors by competitive synthetic or naturally synthesized molecules using some bacteria strains including *V. fischeri*, *Agrobacterium tumefaciens*, *Chromobacterium violaceum*, and *Aeromonas salmonicida*, producing inhibitor proteins which express new functions to cells or by some uncompetitive molecules cause disruption of HSL binding to proteins; (iii) interfering intercellular signaling systems using quorum sensing inhibitors produced by algae, microorganisms, herbs, fruits, spices, and animals (Lazar, 2011; Bai and Vittal, 2014; Machado et al., 2020). Quorum sensing inhibitors as intrinsic ammunition are considered to preserve from treatment to surviving the host cells or hindering the ecological balance (Sampathkumar et al., 2019). Generally, quorum sensing inhibitors are divided into three main classes, namely, AHL analogs, 2 (5H)-furanones, and compositions that are not structurally related to AHLs (Lazar, 2011). However, there are four enzymatic ways to degrade AHL, including AHL acylases (amidohydrolysis) and AHL lactonases (lactone hydrolysis), which hydrolyze the amide bonds of the AHL molecules irreversibly and the HSL rings reversibly; AHL oxidases and AHL reductases (oxidoreduction) alter the activity of AHL but are not able to degrade the AHL molecules (Dong et al., 2000; Uroz et al., 2009; Borges and Simões, 2019). It has repeatedly been shown in several studies that AHL analogs modified the acyl side or lactone ring, or amide moiety by which the lactone moiety is replaced by cyclopentyl or cyclohexanone rings or triazolyldihydrofuranone moiety, interfering biofilm development and indicating biofilm inhibitory action against *Serratia marcescens*, *P. aeruginosa,* and *Burkholderia* spp. (Ishida et al., 2007; Morohoshi et al., 2007; Brackman et al., 2012). Nevertheless, exploring new and natural sources for inhibiting biofilm-related infections would be crucial to be used in clinical and industrial practices. Meanwhile, biological molecules from algal sources are promising agents due to their vast species diversity, habitat they live in, and great biological defense activity compared with their counterparts from other resources (López and Soto, 2019).

## 5 Marine anti-biofilm agents

Recently, some investigations have been focused on natural products with anti-biofilm attributes. Therefore, various bioactive compounds have been identified from marine sponges, including the diterpene alkaloid (−)-ageloxime D 121, derived from *Agelas nakamurai* with prevention of *S. epidermidis* biofilm formation (Hertiani et al., 2010), diterpene, darwinolide 122 from *Dendrilla membranosa* with inhibition of the attachment and biofilm formation of MRSA (von Salm et al., 2016), tryptamine derivative bufotenine 123, from the Mediterranean gorgonian *Paramuricea clavate* with anti-adhesion activity against *Pseudoalteromonas* sp. D41 (Pénez et al., 2011). Sometimes, these natural compounds, such as bufotenine (5) and 1,3,7-trimethylisoguanine (10), two bromoindole derivatives, are more effective than commercial compounds and have more strong anti-adhesion properties against bacteria (Pénez et al., 2011). The marine-based natural anti-biofilm agents are well reviewed by Melander, Basak (Melander et al., 2020). However, some algae species producing inhibitors such as red marine algae, *Delisea pulchra*, synthesizes halogenated furanones as the first quorum sensing inhibitor group which competitively inhibits AHL binding to intracellular receptors and then bacterial colonization and biofilm development. It has happened by preventing the key quorum sensing pathway through bacterial cells as the AHSL regulatory system in Gram-negative bacteria and also AI-2 signaling network in both Gram-negative and Gram-positive species (Manefield et al., 1999; Dong et al., 2000; Borges and Simões, 2019). Moreover, it has been observed that extracts from red seaweeds *Sarcodiotheca gaudichaudii* and *Chondrus crispus* interfere with biofilm formation by *S. enteritidis* (Kulshreshtha et al., 2016). The great anti-bacterial and anti-biofilm activities of methanolic extract of *Padina pavonica* were obtained against *S. aureus*, *Enterococcus faecalis*, *P. aeruginosa*, *Klebsiella pneumonia*, and *B. subtilis* (Makhlof et al., 2023). Attenuation of AHL function of several marine actinomycetes extracts has been discovered against biofilm formation of *Vibrio* spp., hindering quorum sensing signaling by blocking the AHL receptor site of the *LuxR* homologs (You et al., 2007).

### 5.1 Algae

Algae are a very diverse group of predominantly aquatic photosynthetic microorganisms that account for almost 50% of photosynthesis on this planet. Marine algae are classified into two major groups based on their size; seaweeds (macroalgae) and microalgae (Menaa et al., 2021). Seaweeds can be classified into Chlorophyceae (green algae), Phaeophyceae (brown algae), and Rhodophyceae (red algae). Marine algae have been recognized as an alternative resource of natural substances which possess a wide range of biological attributes and health benefits such as antioxidant, antibacterial, antifungal, antiviral, anti-inflammatory, and antidiabetic attributes (Pradhan et al., 2020; Oliyaei et al., 2022). Therefore, algal metabolites have gained considerable attention in the development of functional foods and nutraceutical industries and health products (Oliyaei et al., 2022). In recent years, the anti-biofilm activity of algae extracts and their derivatives has gained much attractive attention which will be reviewed (Table 1).

**Table 1:** Attributes of algal-base anti-biofilm agents.

Inhibitor	Algae	Organism	Effect	Reference
Algae extracts	*W. prolifica*	Gram-positive bacteria (*S. aureus, B. subtilis*, and *Streptococcus* spp.), gram-negative bacteria (*Shigella* spp., *Proteus* spp., and *P. aeruginosa*) and fungi (*A. niger* and *C. albicans*).	Inhibition of biofilm formation and prevention of bacterial adhesion. Antibacterial activities of Phycobiliprotein pigments, such as phycocyanin	Al-TmimiG et al. (2018)
	*A. platensis*	*V. parahaemolyticus* *Ch. Violaceum*, *V. alginolyticus* *A. hydrophila* *P. aeruginosa* *E. coli* *S. aureus*	Reduction of the cell surface hydrophobicity and inhibition of EPS	LewisOscar et al. (2017)
	*Chlamydomonas* spp.	*P. aeruginosa* (PA14 and ATCC 10145)	Reduction of cell surface hydrophobicity and extracellular polymeric substances and biofilm secretion, Acyl homoserine lactone (AHL)-like substances affect quorum sensing-controlled expression, reduction of biofilm morphology and thickness of biofilm by furanones, and the anti-biofilm activity and pathogenicity of *P. aeruginosa* by phycocyanin	Nithya et al. (2014)
	*Synechococcus* spp.	*V. harveyi* and *V. vulnificus*	Inhibition of the initial attachment and interfering in the biofilm structure and formation. Interfere in quorum sensing pathway by blocking the protease and gelatinase	Santhakumari et al. (2016)
	*U. reticulate, S. wightii*, *H. macroloba*, sea grasses: *H. pinifolia*, *C. serullata*	*Pseudomonas* spp., *Flavobacterium* spp., *Bacillus* spp., and *Cytophaga* spp.	Inhibition of biofilm formation mainly related to the functional groups such as hydroxyl and carbonyl groups, fatty acids, and amides I & II.	Prabhakaran et al. (2012)
	225 cyanobacteria microalgae species (*Cercozoa, Charophyta, Chlorophyta, Cryptophyta, Cyanobacteria, Euglenophyta, Glaucophyta, Haptophyta, Miozoa, Ochrophyta, Rhodophyta*, and 2 unknown species)	*C. albicans* and *C. cloacae*	Biofilm inhibition mechanism because of high amount of lipids and polyunsaturated fatty acids (PUFAs) such as docosahexaenoic acid	Cepas et al. (2019)
	*Chlamydomonas reinhardtii*	*E. cloacae, C. albicans,* and *S. aureus*	Inhibition of bacteria growth, quorum quenching, and disruption of biofilm because of active metabolites such as hydrocarbons, phenols, alcohols, and esters and two bioactive compounds;1Heptacosanol and Octadecyl chloride	Ghaidaa et al. (2020)	
	*H. siliquosa*	*S. aureus* MRSA ATCC 33593 and *S. aureus* MRSA NCTC 10442	The strong antimicrobial properties were related to QSI	Busetti et al. (2015)	
	*Halimeda* spp.		Inhibition of bacteria growth and EPS production	Gadhi et al. (2018)	
	*S. wightii*, and *H. gracillis*	Gram-negative bacteria (*E. coli, P, aeruginosa*, and *V. parahaemolyticus*).	Antimicrobial activity against the biofilm formation by gram-negative bacteria, insecticidal activity	Suganya et al. (2019)	
	*C. spongiosus, L. papillosa*, and *C. arabicum*	*Candida* species (*C. krusei, C. glabrata, C. parapsilosi,* and *C. albicans*).	Inhibition of *C. krusei* biofilm formation, reduction in the viability of preformed biofilms	El Zawawy et al. (2020)	
	*Sargassum* spp.	*S. epidermidis*	Inhibition of biofilm formation by *Staphylococcus* spp.	Alreshidi et al. (2023)	
	*U. lactuca* *S. scoparium*, and *P. capillacea*	*S. aureus*	Reduction in the surface hydrophobicity of *S. aureus* and inhibition of initial adhesion and proliferation of *S. aureus* and the biofilm development	Rima et al. (2022)
Bioactive compounds	Furanones derived from *D. pulchra*,		Interfering in gene expression of the AHL and act as quorum sensing inhibitor	Manefield et al. (1999)
	Alginate oligosaccharides derived from *L. hyperborean*	*P. aeruginosa*	Weakening the EPS integrity and decomposition of its structure	Powell et al. (2018)
	Fucoidan F85	*S. mutans* and *S. sobrinus*	Inhibition of the planktonic cell growth	Vishwakarma and Vavilala (2019)
	Fucoidan F85 derived from *F. vesiculosus*	*S. mutans* and *S. sobrinus*	Inhibition of biofilm production and planktonic cell growth	Jun et al. (2018)
	Zeaxanthin	*P. aeruginosa*	Interfering in the gene expression of quorum sensing and prevention of *LasIR* and *RhlIR* quorum sensing system	Gökalsın et al. (2017)
	Lutein derived from *C. pyrenoidosa*	*P. aeruginosa*.	Inhibition of biofilm formation and degeneration of CSH and EPS. Interact with *LasI*, *LasR*, *RhlI,* and *RhlR* taking part in quorum sensing process	Sampathkumar et al. (2019)
	Glycolipid derived from *Shewanella algae*	*B. cereus*, *S. pneumoniae*, *P. aeruginosa*, *E. coli*, *K. pneumoniae*, and *Acinetobacter* spp.	Growth inhibition of clinical bacterial pathogens and disruption of the preformed biofilms	Gharaei et al. (2022)
	Lipid from *S. platensis* extract	*C. albicans*	Anti-biofilm growth activity and anti-biofilm activity	Boutin et al. (2019)
	Lipids from *S. brasiliensis*, *E. acutiformis* and cyanobacteria (*Sphaerospermopsis* spp.)	*K. pneumoniae*, *E. coli, P. aeruginosa*, *E. cloacae, S. aureus*, Coagulase Negative *Streptococcus*, *S. epidermidis*, *C. parapsilosis*, and *C. albicans*	Effective inhibition of biofilm and antivirulence potency	Prasath et al. (2020)
	Palmitic acid derived from *Oscillatoria subuliformis*	*P. aeruginosa*	Reduction of biofilm formation by downregulation of *abaR* gene, reducing N-acyl-homoserine lactone production and interference in quorum sensing system	LewisOscar et al. (2018)
	Phlorotannins from *Ascophyllum nodosum*	*E. coli*	Inhibition of cell proliferation and synthesis of exopolysaccharides	Bumunang et al. (2019)
	Phlorotannins from *Hizikia fusiforme*	*P. aeruginosa*	Reduction of pyocyanin production, disruption of QS, reduction of the motility of bacterial, synthesis of protease, and hemolysin, and suppression of the biofilm formation	Tang et al. (2020)
	Polyphenols from *Sargassum muticum*	*E. coli* and *P. aeruginosa*	Anti-biofilm activity by suppressing the biofilm formation	Puspita et al. (2017)
Green silver nanoparticles	Silver nanoparticles in combination with seaweeds extracts (*U. fasciata*, *Grateloupia* spp., *P. capillacea* and *C. mediterranea*)	*E. coli*, *S. aureus*, *S. faecalis*, *P. aureogenosa* and *V. damsela.*	Anti-biofilm activity	Negm et al. (2018)
	Silver nanoparticle with *Oscillatoria* spp. extract	*S. aureus*, *E. coli*, *P. aeruginosa*, *Citrobacter* spp., *S. typhi*, and *B. cereus*.	Antibacterial activity against pathogen bacteria, strong antibiofilm activity	Adebayo-Tayo et al. (2019)
	Silver nanoparticle using red algae *G. corneum* extract	*C. albicans* and *E. coli*	Antibiofilm efficacy in two stages (prebiofilm and postbiofilm)	Öztürk et al. (2020)
	Silver nanoparticles with *G. corticata*	*K. pneumonia*	Influenced the protein responsible for EPS production and disrupting the protective layer in biofilm	Rajivgandhi et al. (2020)
	Silver nanoparticles with *Sargassum myriocystum*	*P. aeruginosa,* and *S. epidermidis*	Biofilm inhibition at MIC due to the inhibitory effect on gene expressions related to motility and biofilm formation	Balaraman et al. (2020)
	Silver nanoparticles using *Spirogyra* extract	*S. aureus* and *Acinetobacter baumannii*.	Lethal to biofilm-forming bacteria and inhibiting biofilm development	Danaei et al. (2021)
	Iron oxide nanoparticles prepared using *S. vulgare*, *U. fasciata* and *J. rubens* extract	Gram-positive and Gram-negative bacteria	Antibiofilm efficiency on steel and wood surfaces	Salem et al. (2020)
	Silver nanoparticles with *S. angustifolium*		Increasing lethality with increasing concentration	Bita et al. (2015)
	Silver nanoparticles with *Urospora* spp.		Inhibitory activity against pathogen bacteria	Suriya et al. (2012)
	Silver nanocomposite capped with k-carrageenan	*S. aureus* and *P.aeruginosa*	Reduction in the growth of biofilm forming bacteria	Goel et al. (2019)
	Silver nanoparticles capped with k-carrageenan	*C. albicans* and *C. glabrata*	Cell membrane penetration and interaction with extracellular matrix of biofilms	Gupta et al. (2021)
Dental resin	Dental acrylic resin containing seaweeds *U. pinnatifida*	*S. mutans*	Reduction in the colony counts of microorganisms and antibiofilm activity	Pourhajibagher et al. (2019)
	Silver nanoparticles with *U. lactuca*	*S. mutans*	Reduction of virulence genes expression and biofilm formation ability	Pourhajibagher et al. (2021)
	Nanocomposite with *S. muticum* extract containing Zn and CuO nanoparticles	*P. mirabilis*, *P. aeruginosa*, *S. aureus*.	Reduction of biofilm production and inhibition of adhesion	Sadek et al. (2019)

#### 5.1.1 Algae extract

Some researchers have investigated the anti-biofilm activity of algae extracts. The blue-green algae *Westiellopsis prolifica* extract showed anti-biofilm activity potential against gram-positive bacteria (*S. aureus*, *B. subtilis*, and *Streptococcus* spp.), gram-negative bacteria (*Shigella* sp., *Proteus* sp., and *P. aeruginosa*), and fungi (*Aspergills niger* and *Candida albicans*). It was reported that the *W. prolifica* extracts were more effective on gram-negative bacteria, and the crude acetone extract showed the highest biofilm inhibition against *Shigella* sp. According to the Congo red agar method, the *W. prolifica* acetone extract exhibited significant anti-biofilm activity at 50 μL, while hexane extract was less active. The *W. prolifica* extract can inhibit bacterial biofilm formation and prevent bacterial adhesion, which depends on its antibacterial components such as phycobiliprotein pigments such as phycocyanin, phycoerythrin, and allophycocyanin (Al-TmimiG et al., 2018). The methanolic *Arthrospira platensis* extract at a concentration of 100 ng mL^−1^ also prevented the biofilm formation of *V. parahaemolyticus* (90%), *Chromobacterium violaceum* (89%), and *V. alginolyticus* (88%). Moreover, spirulina extract lowered the cell surface hydrophobicity (CSH) of *A. hydrophila*, *E. coli,* and *S. aureus* impressively and also exhibited extracellular polymeric substances (EPS) inhibition of approximately 50–88% for *P. aeruginosa*, *E. coli*, *S. aureus,* and *A. hydrophila* (LewisOscar et al., 2017). Accompanying quorum sensing, CSH and EPS play a significant role in attachment and biofilm forming ability of bacteria. The structural integrity and stability of biofilm is influenced by EPS. Surface adhesion of bacteria and enhancement of biofilm mass are also strongly affected by CSH. Therefore, CSH diminish can intercept initial attachment and reduce early stage biofilms, thereby reducing the bacterial communities in biofilm and biofilm-related disorders (LewisOscar et al., 2017). Similarly, *Chlamydomonas* sp. extract can decrease CSH and biofilm secretion of *P. aeruginosa*. *Chlamydomonas* extract has some Acyl homoserine lactone (AHL)-like substances that affect quorum sensing-controlled expression. Moreover, reduction of the biofilm morphology, thickness by furanones, and anti-biofilm activity and pathogenicity of *P. aeruginosa* by phycocyanin are reported (Nithya et al., 2014).

In addition, the crude extract of *Synechococcus* sp. possesses anti-biofilm and anti-quorum sensing activity against aquatic bacterial pathogens, such as *V. harveyi* and *V. vulnificus*, and significantly decreased the biofilm formation (approximately 71 and 84%) and EPS secretion (approximately 66 and 68%). It has been reported that this extract successfully inhibited the initial attachment, interrupted the biofilm structure, and therefore prevented biofilm formation. Moreover, the *Synechococcus* extract can interfere in the quorum sensing pathway by blocking the protease and gelatinase, which were positively regulated by the quorum sensing. The anti-quorum sensing activity of the *Synechococcus* extract was related to the presence of hexadecanoic acid in the extract, and molecular docking revealed its potential to antagonistic binding to the AHL receptor protein (Santhakumari et al., 2016).

Several researchers reported that polar and non-polar extracts from brown algae, including *Sargassum vulgare*, *Padina* sp., *S. furcatum*, and *Dictyota* sp., and red algae, such as *Asparagopsis taxiformis*, *Pterocladiella capillacea*, *Spyridia aculeate*, and *Peyssonnelia capensis,* exhibited anti-QS activity (Dahms and Dobretsov, 2017). It has also been reported that different extracts from seaweeds such as *U. reticulate*, *Sargassum wightii*, *Halimeda macroloba*, and sea grasses, such as *Halodule pinifolia* and *Cymodocea serrulata,* exhibited anti-biofilm formation activity against *Pseudomonas*, *Flavobacterium*, *Bacillus*, and *Cytophaga* species. This inhibitory activity was related to the main functional groups, including hydroxyl, amino, carbonyl, and phosphoryl functionalities, aliphatic extracts (fatty acids), and NH2 (amide I and II) extracts (Prabhakaran et al., 2012). Similarly, Cepas and López (Cepas et al., 2019) investigated the anti-biofilm activity of 675 extracts (hexane, ethyl acetate, and methanol) obtained from 225 cyanobacteria and microalgae species (11 phyla including *Cercozoa*, *Charophyta*, *Chlorophyta*, *Cryptophyta*, *Cyanobacteria*, *Euglenophyta*, *Glaucophyta*, *Haptophyta*, *Miozoa*, *Ochrophyta*, and *Rhodophyta* and two unknown species). The results showed the highest biofilm inhibition rates of algae extracts against *Candida albicans* and *Enterobacter cloacae*. Among various microalgae, *Chlorophyta* and *Charophyta* showed high minimal biofilm inhibitory concentration (MBIC) values due to their ability to produce and accumulate high amounts of bioactive compounds; in particular, lipids and polyunsaturated fatty acids (PUFAs) such as docosahexaenoic acid (Pradhan et al., 2020) and eicosapentaenoic acid (Cepas et al., 2021) possess antimicrobial and anti-biofilm activities. In addition, some algae groups exhibited particular anti-biofilm activity against certain bacteria. For instance, biofilm formation in *Enterobacter cloacae* was most susceptible to *Rhodophyta* species extracts, while biofilm formation in *Candida albicans* was influenced by extracts from *Cryptophyta*, *Euglenophyta*, and *Glaucophyta*. These observations were related to the differences in antibacterial secondary metabolites such as circular or linear lipopeptides, amino acids, fatty acids, macrolides, and amides. Similarly, the extract from green microalgae *Chlamydomonas reinhardtii* reduced the biofilm formation of *S. aureus* by approximately 89%. The presence of bioactive substances in algae extracts such as hydrocarbons, phenols, esters, 1-Heptacosanol, and Octadecyl chloride prevent the biofilm formation via the inhibition of bacteria growth, quorum quenching, and disruption of biofilm (Ghaidaa et al., 2020).

The hexane: ethyl acetate extract of brown alga *Halidrys siliquosa* from Ireland at 1.25 mg ml^−1^ and 5 mg ml^−1^ concentration had inhibition against biofilms by bacterial human pathogens such as *S. aureus* MRSA ATCC 33593 and *S. aureus* MRSA NCTC 10442, respectively (Busetti et al., 2015). Gadhi, El-Sherbiny (Gadhi et al., 2018) prepared different types of red algae *Halimeda* sp. extracts from surface and whole tissue and also prepared dried and fresh algae using methanol and hexane. The authors obtained various inhibitory activities in different approaches. The results showed that dried *Halimeda* sp. extracts, especially methanolic extract, had higher anti-proliferative activity and EPS production compared with fresh algae extracts, while the fresh *Halimeda* sp. extracts exhibited better inhibition against the biofilm formation, which might be related to differences in their metabolites due to the loss of volatile compounds, such as terpenoids and fatty acids, during the drying process. Moreover, the highest biofilm formation inhibitory was obtained in the hexane surface algae extract, which might be attributed to the presence of butanamide and piperazine derivatives. In similar investigation, Suganya and Ishwarya (Suganya et al., 2019) revealed that the ethanol extracts from brown seaweed *S. wightii* and green seaweed *Halimeda gracilis* had significant anti-biofilm formation activity up to 40–75% against gram-negative bacteria (*E. coli*, *P. aeruginosa*, and *V. parahaemolyticus*). Moreover, the anti-QS activity of red seaweed *Asparagopsis taxiformis* extract was related to active fraction of 2-dodecanoyloxyethanesulfonate (C_14_H_27_O_5_S) (Jha et al., 2013). Other red macroalgae extracts such as *Chondrus crispus* and *Sarcodiotheca gaudichaudii* also showed the downregulation of QS gene *sdiA* expression, limiting the bacterial motility, reduction of virulence factors, and biofilm formation of *Salmonella enterica* (Kulshreshtha et al., 2016). In addition, three AHL inhibitors were detected from the Korean red algae *Ahnfeltiopsis flabelliformis* (Kim et al., 2007). Batista et al. (2014) used polar (water: methanol) and non-polar (dichloromethane) extracts of different macroalgae as QS inhibitors. The authors reported that 20 polar extracts interrupted the QS of AHL producer and reporter *Chromobacterium violaceum* CV017 with an MIC value ranging from 0.28 μg ml^−1^ (*Ulva fasciata*) to 189 μg ml^−1^ (*Codium* sp.). The MIC value of non-polar extracts ranged from 69 μg ml^−1^ (*Sargassum furcatum*) to 2730 μg ml^−1^ (*Peyssonnelia capensis*).

In addition, *Candida krusei*, is one of the highest biofilm producer among *Candida* isolates which was sensitive to the acetone extract of brown seaweed *Cladostephus spongiosus* (El Zawawy et al., 2020). El Zawawy et al. (2020) investigated the inhibitory effect of different organic extracts (acetone, ethanol, and methanol) of brown alga *Cladostephus spongiosus*, red alga *Laurencia papillosa*, and green alga *Codium arabicum,* which were collected from the coastal region of Hurghada in Egypt against *Candida*. Among all extracts, acetone extract of *C. spongiosus* exhibited the highest inhibitory activity against different *Candida* species (*C. krusei*, *C. glabrata*, *C. parapsilosis,* and *C. albicans*). Acetone extract of *C. spongiosus* was composed of three main fractions, namely, 4-hydroxy-4-methyl-2-pentanone, n-hexadecenoic acid, and phenol, 2-methoxy-4-(2-propenyl), which were detected by GC–MS and were not identified in other extracts. The anti-biofilm ability of acetone extract of *C. spongiosus* attributed to these fractions and caused downregulation of hyphal-specific genes, hyphal wall protein 1 (HWP1), agglutinin-like protein 1 (ALS1), and fourth secreted aspartyl proteinase (SAP4). The authors can intercept biofilm formation and eliminate the existing biofilm. Moreover, the methanol/water extract of brown seaweed *Sargassum* sp. exhibited remarkable anti-biofilm activity (82.35%) against *S. epidermidis* at 12.5 mg ml^−1^. The extract was mainly consisting of 2-pentadecanone, 6,10,14-trimethyl-, hexadecenoic acid, methyl ester, n-hexadecanoic acid, 1,2-benzenedicarboxylic acid, and mono (2-ethylhexyl) ester, and its anti-biofilm activity was due to the chemical interaction between extract substances and some targeted receptors (Alreshidi et al., 2023).

Furthermore, the green *Ulva lactuca* extract was considered as a renewable source of new active anti-biofilm agents. Investigation reported that the polar and non-polar extracts of green seaweeds such as *Caulerpa racemose*, *Codium* spp., and *Ulva (Enteromorpha) fasciata* had anti-QS activity (Dahms and Dobretsov, 2017). Rima and Trognon (Rima et al., 2022) explored the biofilm formation inhibition activity of extracts from various macroalgae, including green alga *U. lactuca*, brown alga *Stypocaulon scoparium*, and red alga *Pterocladiella capillacea* toward *P. aeruginosa*. Among different seaweeds, green algae extracts (cyclohexane and ethyl acetate) showed better inhibitory effect against bacterial adhesion rather than others. The same prevention was observed against *S. aureus* biofilm formation in all these three seaweed extracts (Rima et al., 2022).

#### 5.1.2 Algae bioactive compounds as anti-biofilm agents

##### 5.1.2.1 Algal polysaccharides

Seaweeds are good sources of carbohydrates whose content is from 5 to 75% (w/w, DW) based on the age, species, period, and harvesting site. Seaweed polysaccharides are divided into sulfated and non-sulfated forms. Green seaweeds are rich in ulvan, while brown seaweeds contain different types of polysaccharides in their cell walls, including alginate, laminaran, and fucoidan. Moreover, red macroalgae are identified by their carrageenans, agars, xylogalactans, sulphated galactans, xylans, porphyran, and floridean starch. Several research studies confirmed the biological attributes of algal carbohydrates and their derivatives (Hentati et al., 2020). The pharmacological effects of polysaccharides are related to their chemical structure, molecular weight, linkage types, and monosaccharides (Xu et al., 2017).

The first anti-biofilm algae natural substance was identified by Manefield et al. (1999), who reported that a halogenated furanone, derived from red alga *Delisea pulchra*, interfered in the gene expression of acylated homoserine lactone (AHL) and act as a quorum sensing inhibitor. Regarding the anti-biofilm activity of algae polysaccharides, alginate oligosaccharides isolated from the brown seaweed *Laminaria hyperborean* were able to decline *P. aeruginosa* biofilm biomass by weakening the EPS integrity and decomposing its structure (Powell et al., 2018). Similarly, fucoidan F85 at concentration above 250 μg ml^−1^ fully suppresses the *Streptococcus mutans* and *S. sobrinus* biofilm formation and inhibits the growth of planktonic cells (Vishwakarma and Vavilala, 2019). Some researchers have demonstrated that fucoidan is able to act as anti-biofilm forming dental plaque bacteria. The fucoidan F85 obtained from *Fucus vesiculosus* entirely ceased the biofilm production and planktonic cell growth of *S. mutans* and *S. sobrinus* at concentrations higher than 250 μg ml^−1^ (Jun et al., 2018).

##### 5.1.2.2 Algal carotenoids

Carotenoids are lipid-soluble pigments with health benefits for human daily diets. The major carotenoids found in algae are astaxanthin, fucoxanthin, β-carotene, lutein, siphonaxanthin, zeaxanthin, violaxanthin, neoxanthin, and antheraxanthin (Generalić Mekinić et al., 2023). Recent studies were conducted to evaluate the anti-biofilm and anti-quorum sensing activity of algae carotenoids. For instance, zeaxanthin can interfere in the gene expression of quorum sensing network regulates of *P. aeruginosa* and is able to prevent *LasIR* and *RhlIR* quorum sensing system by approximately 67.8 and 66.1%, respectively, at 12 μM concentration (Gökalsın et al., 2017). Sampathkumar et al. (2019) investigated that lutein was derived from *Chlorella pyrenoidosa* to combat the biofilm formation of *P. aeruginosa*. The authors revealed that lutein exhibited strong inhibition of biofilm formation and degeneration of CSH and EPS at a concentration of 20 μg ml^−1^. According to the docking screening, the anti-quorum sensing activity of lutein was due to its ability to interact with four proteins, including *LasI*, *LasR*, *RhlI*, and *RhlR,* taking part in the quorum sensing process through biofilm production. The highly conjugated double bond structure of lutein with phenolic and polyphenolic function groups such as gallic acid, catechin, and tannic acid provide some binding sites to interact with quorum sensing proteins. The strong hydrophobic interactions between lutein and *RhlI* protein were established. Lutein also decreased gene expression of las and *rhl* in *P. aeruginosa* PAO1, which confirmed the lutein quorum sensing inhibitory effect (Sampathkumar et al., 2019). Moreover, the glycolipid biosurfactant isolated from *Shewanella algae* was able to reduce bio-film production by *B. cereus* (83%), *S. pneumoniae* (53%), *P. aeruginosa* (92%), *E. coli* (64%), *K. pneumoniae* (87%), and *Acinetobacter* spp. (72%) (Gharaei et al., 2022).

##### 5.1.2.3 Algal lipids

Algae are rich in essential fatty acids, especially polyunsaturated fatty acids (PUFAs) such as docosahexaenoic (DHA), eicosapentaenoic (EPA), and linoleic (LA) (Chen et al., 2023). Moreover, algal sterols (fucosterol, clionasterol, isofucosterol, and cholesterol) are recognized as nutritional and important components with several health benefits such as anticancer, antioxidant, antiobesity, and antiviral activities. Like other bioactive compounds from algae, their lipid content and fatty acid profile are varied and depend on algae species, season and harvesting time, weather, and geographical location (Hentati et al., 2020).

Recently, it has confirmed that the *S. platensis* lipid-rich extract exhibited 80% inhibition of biofilm growth against *C. albicans* after 24 h at 0.2 mg ml^−1^, while encapsulation of *S. platensis* lipid extracts by copper-alginate nanocarriers represented adequate anti-biofilm activity (50% inhibition at 0.1 mg ml^−1^). *Spirulina* lipid extract was mainly containing the γ-linolenic acid and linoleic acid affecting its anti-biofilm activity (Boutin et al., 2019). Cepas and Gutiérrez-Del-Río (Cepas et al., 2021) reported that microalgae (*Scenedesmus brasiliensis* and *Enallax acutiformis*) and cyanobacteria (*Sphaerospermopsis* spp.) lipids exhibited antibacterial and anti-biofilm activity against gram-negative bacteria, gram-positive bacteria, and fungi (*K. pneumoniae*, *E. coli*, *P. aeruginosa*, *E. cloacae*, *S. aureus*, Coagulase-negative *Streptococcus*, *S. epidermidis*, *C. parapsilosis*, and *C. albicans*). The lipid-rich extracts mainly comprised sulfoquinovosyldiacylglycerol, monogalactosylmonoacylglycerol, sulfoquinovosylmonoacylglycerol, α-linolenic acid, hexadeca-4,7,10,13-tetraenoic acid (HDTA), palmitoleic acid, and lysophosphatidylcholine. However, the anti-biofilm activity of *Scenedesmus* strain extracts was related to the α-linolenic acid. α-linolenic acid is the precursor to the important long chain omega-3 fatty acids, eicosapentaenoic acid (EPA), and docosahexaenoic acid (DHA). It has been reported that the pure commercial α-linolenic acid is able to inhibit the biofilm formation by *P. aeruginosa*, *S. aureus*, and *C. albicans* at 64 mg L^−1^. Moreover, it has been revealed that fatty acids such as palmitic acid can reduce approximately 54% of *Candida* biofilm production at concentration of 500 mg L^−1^ due to the induction of the reactive oxygen species system (Prasath et al., 2020), while palmitic acid derived from the *Oscillatoria subuliformis* extract had 54% bio-film inhibitory activity against *P. aeruginosa* (LewisOscar et al., 2018). This funding revealed that the palmitic acid leading to a reduction in biofilm formation by down regulation of abaR gene, reducing N-acyl-homoserine lactone production, resulting in interfering with the quorum sensing system (Cepas et al., 2021).

##### 5.1.2.4 Algal phlorotannins

Phlorotannins are polyphenolic compounds mostly found in brown algae, with the structural role in cell walls and formed by the polymerization of phloroglucinol unites (Lemesheva et al., 2023). The highest amount of phlorotannins is found in the brown algae *Fucus* sp. reaching up to 12% of dry weight. Phlorotannins exhibit a wide range of molecular weight (126 Da to 650 kDa), although the majority of phlorotannins exhibit low-molecular weight in the range of 10–100 kDa. The molecular weight of phlorotannins depends on algae species, size, geographic region of growth, environmental condition (light, temperature, and water salinity), nutrient level, season, and extraction techniques (Pradhan et al., 2022). These phenolic compounds are subclassified as phloroglucinol, eckol, phlorofucofuroeckol A, phlorofucofuroeckol B, 2-phloroeckol, dieckol, 6.6-bieckol, and 8.8-bieckol (Lemesheva et al., 2023). Phlorotannins can be divided into four main groups, according to the structural linkage types between phloroglucinol units: phlorethols and fuhalols (with ether linkage), fucols (with phenyl linkage), fucophlorethols (with both ether and phenyl linkages), and phloreckols (with dibenzodioxin linkage) (Li et al., 2017).

Current knowledge of phlorotannins in brown seaweeds revealed their antibiofilm and anti-fouling activities. Their biofilm inhibition is attributed to QS suppression and prevention of bacterial adhesion (Besednova et al., 2020). Phlorotannins extracted from seaweed *Ascophyllum nodosum* exhibited anti-biofilm activity against two *E. coli* strains (serotypes O113:H21 and O154:H10) through the inhibition of cell proliferation and synthesis of exopolysaccharides in *E. coli* (Bumunang et al., 2019). Similarly, the antibiofilm effect of enzymatic extraction of polyphenols derived from *Sargassum muticum* was reported which might be related to their phlorotannins (Puspita et al., 2017). Recently, the reduction of the production of virulence factor, anti-QS, and anti-biofilm activity of phlorotannins from *Hizikia fusiforme* against *P. aeruginosa* were reported. Phlorotannins remarkably lowered pyocyanin production, leading to the disruption of QS systems, therefore decreasing the violacein production in *Chromobacterium violaceum* 12,472, which is regulated by the QS system. Moreover, phlorotannins reduced the bacterial motility, synthesis of protease, hemolysin, and suppression of the biofilm formation of *P. aeruginosa* (Tang et al., 2020).

### 5.2 Other applications of marine-based anti-biofilm agents

One of the possible approaches to combat biofilm formation is controlling the bacteria growth. As silver has been recognized as excellent antimicrobial agent, several studies have been focused on green synthesis of silver nanoparticles with plants and algae extracts (Negm et al., 2018). For instance, Negm and Ibrahim (Negm et al., 2018) reported that green synthesized silver nanoparticles in combination with seaweed extracts (*Ulva fasciata*, *Grateloupia* spp., *Pterocladiella capillacea,* and *Corallina mediterranea*) have antimicrobial and anti-biofilm potential against *E. coli*, *S. aureus*, *S. faecalis*, *P. aureogenosa,* and *V. damsela*. Moreover, Adebayo-Tayo et al. (2019) synthesize antibacterial and anti-biofilm activities of silver nanoparticles with the *Oscillatoria* spp. extract, which possessed significant inhibition against *S. aureus*, *E. coli*, *P. aeruginosa*, *Citrobacter* spp., *S. typhi*, and *B. cereus*. Similarly, biosynthesized silver nanoparticles using the red alga *Gelidium corneum* extract had anti-biofilm activity against *C. albicans* and *E. coli*. The highest inhibition was observed in prebiofilm treatment (0.51 μg ml^−1^) with 81% reduction, while it was 73% for post-biofilm treatment (2.04 μg ml^−1^) (Öztürk et al., 2020). It had reported that silver nanoparticles fabricated by Gracilaria corticata possess maximum anti-bacterial and anti-biofilm potential (88%) against *K. pneumonia* at 50 μg ml^−1^ and 100 μg ml^−1^, respectively (Rajivgandhi et al., 2020).

Similarly, green silver nanoparticles prepared with *Sargassum myriocystum* aqueous extract had antimicrobial and anti-biofilm activity against clinical pathogens such as *P. aeruginosa* and *S. epidermidis* with the inhibition of 55.49 and 48.34% at 50 μg ml^−1^, respectively (Balaraman et al., 2020). Danaei and Motaghi (Danaei et al., 2021) also synthesize silver nanoparticles using *Spirogyra* algae extract with effective anti-biofilm activity against *S. aureus* and *Acinetobacter baumannii*. Moreover, biosynthesized iron oxide nanoparticles were prepared using *Sargassum vulgare*, *Ulva fasciata,* and *Jania rubens* aqueous extract to investigate their biofilm inhibitory activity against gram-positive and gram-negative bacteria. Among different seaweeds, the best anti-biofilm activity was observed in *S. vulgare*, *U. fasciata*, and *J. rubens* (Salem et al., 2020). In close examination, silver nanoparticles were fabricated using *Sargassum angustifolium* (Bita et al., 2015), *Urospora* spp. (Suriya et al., 2012), and *Sargassum wightii* (Singaravelu et al., 2007). The novel biodegradable silver nanocomposite capped with k-carrageenan (Goel et al., 2019) and anti-biofilm silver nanoparticles capped with k-carrageenan (Gupta et al., 2021) exhibited anti-biofilm attributes successfully against *S. aureus* and *P. aeruginosa* (Goel et al., 2019) and *C. albicans* and *C. glabrata* (Gupta et al., 2021), respectively. However, in these studies, mostly the anti-biofilm and antibacterial activities of silver nanoparticles were discussed.

Similarly, Marzban et al. (2022) developed new antibiofilm copper nanoparticles (CuNPs) with water-soluble polysaccharides from brown seaweed, *S. vulgare* (SPs). Water-soluble *S. vulgare* polysaccharides contained fucoidan and laminaran and mainly consisted of fucose and manose (approximately 50% w/w). According to the authors’ reports, Sps-CuNPs had the highest biofilm inhibition against *S. aureus* MRSA and MSSA at 100 and 50 μg/mL, respectively, while the MIC value against *S. aureus* MRSA and MSSA was approximately 250 and 150 μg/mL, respectively. The anti-biofilm activity of Sps-CuNPs was related to the high affinity of copper ions to biomolecules, such as proteins and peptides throughout carboxyle, amine, and sulfide groups and its ability to disrupt the biofilm integrity.

Moreover, treatment with orthodontic resin contains anti-biofilm agents, which has increased in recent years. Dental plaque or biofilm is characterized as a community of microorganisms embedded in a matrix of polymers that develop on tooth surface (Zhang et al., 2020). These biofilms have high tolerance to antibiotics and provide poor antibiotic penetration (Pourhajibagher et al., 2021). In addition, dental acrylic resin containing seaweeds *U. pinnatifida* (Pourhajibagher et al., 2019) and *U. lactuca* (Pourhajibagher et al., 2021) exhibited dose-dependent anti-biofilm activity against *S. mutans*. The researchers revealed that *U. lactuca* in combination with antimicrobial photodynamic therapy (aPDT) technique lower the gene expression of gtfB, gtfC, and gtfD as the virulence factors in *S. mutans*, subsequently interfering in biofilm formation (Pourhajibagher et al., 2021). Sadek and Farrag fabricated the anti-biofilm nanocomposite by *S. muticum* extract containing Zn and CuO nanoparticles, which were able to reduce the biofilm formation and adhesion of *Proteus mirabilis*, *P. aeruginosa*, and *S. aureus*.

## 6 Conclusion

Several microbial infections are associated with the biofilm formation. A large number of microbial species tend to communicate with others, interact with their surrounding environment, and display multicellularity, resulting in biofilm formation. Communication of microbial cells is dependent on their cell density through quorum sensing signaling phenomenon mediated by the production and secretion of AIs. The involvement of quorum sensing signaling molecules in biofilm formation has been frequently reported, which is responsible for a variety of infections, food spoilage, biofouling, and biocorrosion. However, quorum sensing systems can be applied in expanding quorum sensing inhibitors as an alternative strategy in the inhibition of particular phenotypes expression blocking cell-to-cell communication causing to control the biofilm propagation and can be of great value in the future treatment of bacterial infections. Meanwhile, natural products are an interesting substance to combat with bacterial biofilm and related infections. The results obtained by numerous investigations performed over the last decade promoted the use of marine and algae extracts and their metabolites as anti-biofilm agents. Various algae extracts inhibited the bacterial adhesion, biofilm formation, and biosynthesis of quorum sensing key enzymes. All the above studies demonstrated that marine natural products possess anti-microbial and anti-biofilm activity toward a large number of pathogens.

Experimenting with a combination of extracts or biopolymers in silver nanoparticles or dental composites also provides anti-bacterial and anti-biofilm activities and is promising for the development of anti-biofilm novel formulations. However, some studies are needed to identify the role of algae substances in anti-biofilm attributes of silver nanoparticles and composites. Moreover, the effect of various microalgae and macroalgae extracts and their other metabolites, such as pigments, are recommended. In addition, *in vivo* studies and clinical trials are necessary for treating human infections.

## Author contributions

AB and NO established this concept, searched the literature, and wrote the first draft of this manuscript. MM-N improved and revised the manuscript. All authors contributed to the article and approved the submitted version.
